# An interval of clinically silent gastrointestinal bleed in dysautonomic spinal cord injury: a case report

**DOI:** 10.1186/s12883-023-03114-9

**Published:** 2023-02-14

**Authors:** Theodore E. Margo, Preston R. McMullin, Firas Kaddouh

**Affiliations:** 1 Long School of Medicine, University of Texas Health Science Center at San Antonio, San Antonio, TX USA; 2 Neurosurgery Department, University of Texas Health Science Center at San Antonio, San Antonio, TX USA

**Keywords:** Spinal cord injury, Gastrointestinal bleed, Dysautonomia

## Abstract

**Background:**

Gastrointestinal bleed (GIB) has high incidence in traumatic spinal cord injured (tSCI) patients and can frequently be life-threatening, especially early post-injury. Several risk factors often compound bleeding risk, some are unique to this patient population. Normally, clinical suspicion for GIB arises from symptoms like coffee-ground emesis, hematemesis, melena or even hematochezia. A hemoglobin drop may be a late sign. Due to tSCI, however, patients often experience neurogenic bowels and dysautonomia, which may delay symptom presentation and complicate timely diagnosis of GIB. We report a case of an almost clinically silent GI bleed in the context of acute cervical tSCI.

**Case presentation:**

A 21-year-old female presented with cervical cord transection at C-7 in the setting of motor vehicle rollover, for which surgical decompression was performed. During the acute injury phase, she also received a 10-day course of dexamethasone for symptomatic COVID-19 pneumonia. Two weeks after injury, she underwent percutaneous endoscopic gastrostomy (PEG) placement which demonstrated normal gastric and duodenal anatomy. One week later, a large spike (10x) in blood urea nitrogen: creatinine (BUN: Cr) ratio raised concern for GIB, but hemoglobin remained stable, and stool color remained unchanged. The following day, a gastroenterology consult was requested under increased suspicion of GIB from a sudden 3.5 g/dL hemoglobin drop. The patient received blood transfusion and pantoprazole. An upper endoscopy was performed, revealing three small duodenal ulcers. Melanotic stool ensued afterwards.

**Conclusions:**

Due to dysautonomia, clinical presentation of GIB can be significantly delayed in the tSCI patient population, leaving them vulnerable to succumb to illness. This case illustrates the possibility of an interval in which the patient was bleeding, with the sole indicator being an elevated BUN. Our case calls for closer monitoring of and vigilance for tSCI patients, and possibly employment of different strategies to reduce the incidence and enhance early detection of GIB in tSCI patients to subsequently decrease the morbidity and mortality associated with it.

## Background

Gastrointestinal bleed (GIB) complicates acute traumatic spinal cord injury (tSCI) in up to 22% of patients and can pose a significant risk to their morbidity and mortality [1–3]. The exact mechanism for duodenal and gastric ulceration in this cohort is not well understood, but many hypothesize an underlying risk of neurogenic basis involving unchecked parasympathetic stimulation resulting from absent sympathetic input [4, 5]. Those with cervical and high thoracic spinal cord injuries commonly experience autonomic dysreflexia at an incidence of approximately 90% in complete injury and 27% in incomplete injury. Such pervasive autonomic dysreflexia suggests a majority of tSCI patients have little to no sympathetic input to the gastrointestinal tract, and thus are at increased risk for posttraumatic gastroduodenal ulceration [6, 7]. Though dysautonomia is typically recognized as a complication of chronic tSCI, it also occurs in the acute phase of injury [8], though less frequently, further increasing risk of upper GIB. Besides dysautonomia, several additional risk factors exist for GIB in this patient cohort including acute stress due to trauma, prophylactic (or sometimes therapeutic) anticoagulation, steroid administration, history of alcohol abuse, and mechanical ventilation [2, 6, 9]. Regardless of how great dysautonomia’s role is in the etiology of GIB in tSCI, it can certainly hinder or even mask its clinical presentation. Thus, traditional symptomatic presentation of gastrointestinal hemorrhage such as coffee-ground emesis, hematemesis, melena, and hematochezia can be significantly delayed and may impede timely and lifesaving interventions.

We report a case of acute cervical tSCI in a 21-year-old woman who developed a clinically silent and life-threatening GIB in the context of tSCI associated dysautonomia. This case illustrates frame-by-frame an interval in which bleeding occurred without traditional clinical indicators but was suggested only by an acute spike in blood urea nitrogen (BUN) value and BUN: Creatinine ratio. The initial absence of clinical evidence for GIB in our patient highlights the insidious presentation of a grave complication of tSCI where even clinical hypervigilance may not be sufficient.

## Case presentation

A 21-year-old previously healthy Hispanic woman presented to the hospital with acute cervical spinal cord transection at C7 level after involvement in a high-speed motor vehicle rollover. She was intubated on arrival and placed on mechanical ventilation, then underwent early surgical decompression with C6 laminectomy and C4-T2 posterior spinal fusion. She was started on gastrointestinal prophylaxis with daily famotidine, and on enoxaparin postoperatively per standards of care for venous thrombotic event prophylaxis. Notably, she was positive for SARS-CoV-2 virus (COVID-19) on admission, and one week post admission, she became symptomatic with COVID-19 pneumonia, for which she received a 10-day course of dexamethasone.

**Fig. 1:**
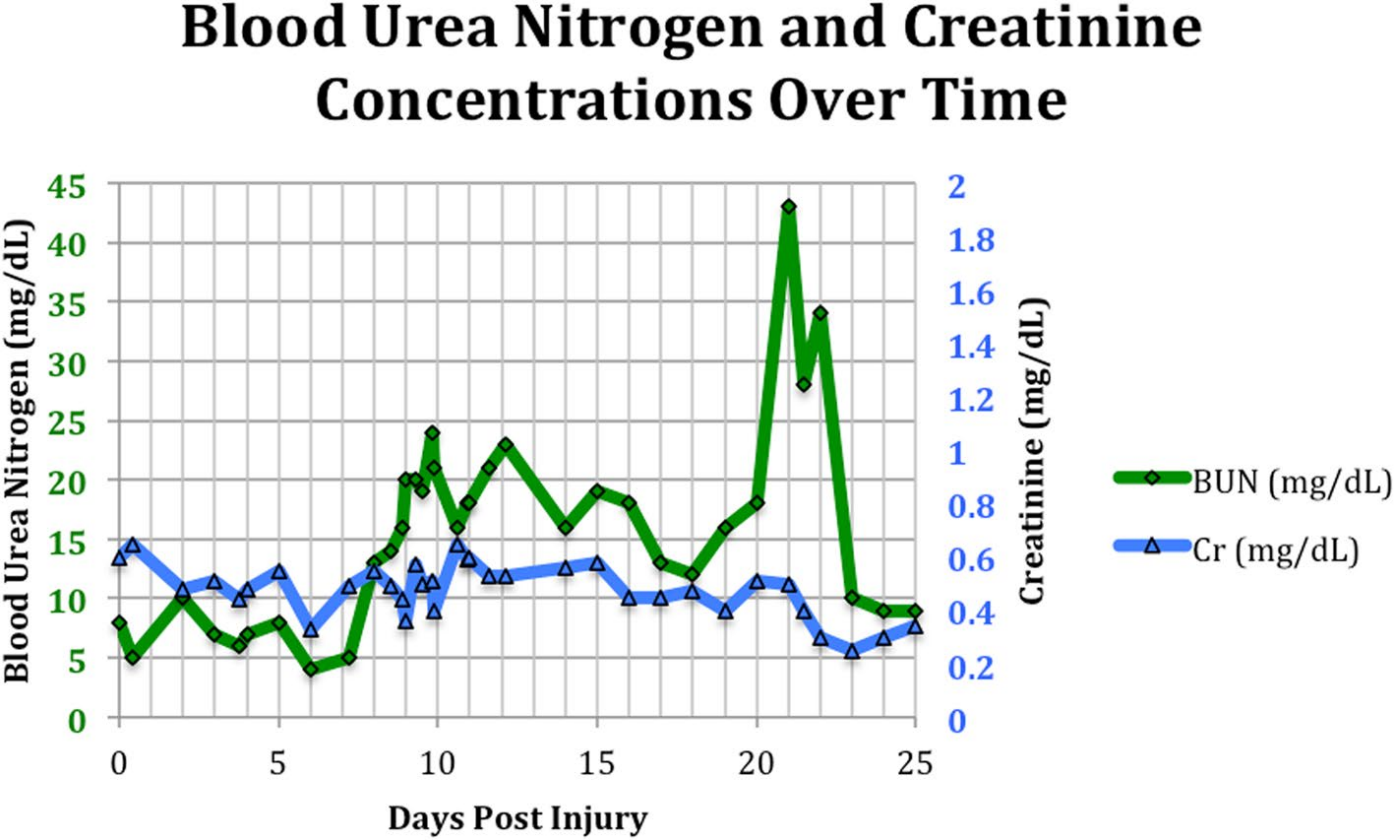
Line graph of blood urea nitrogen and creatinine concentrations during ICU stay

**Fig. 2:**
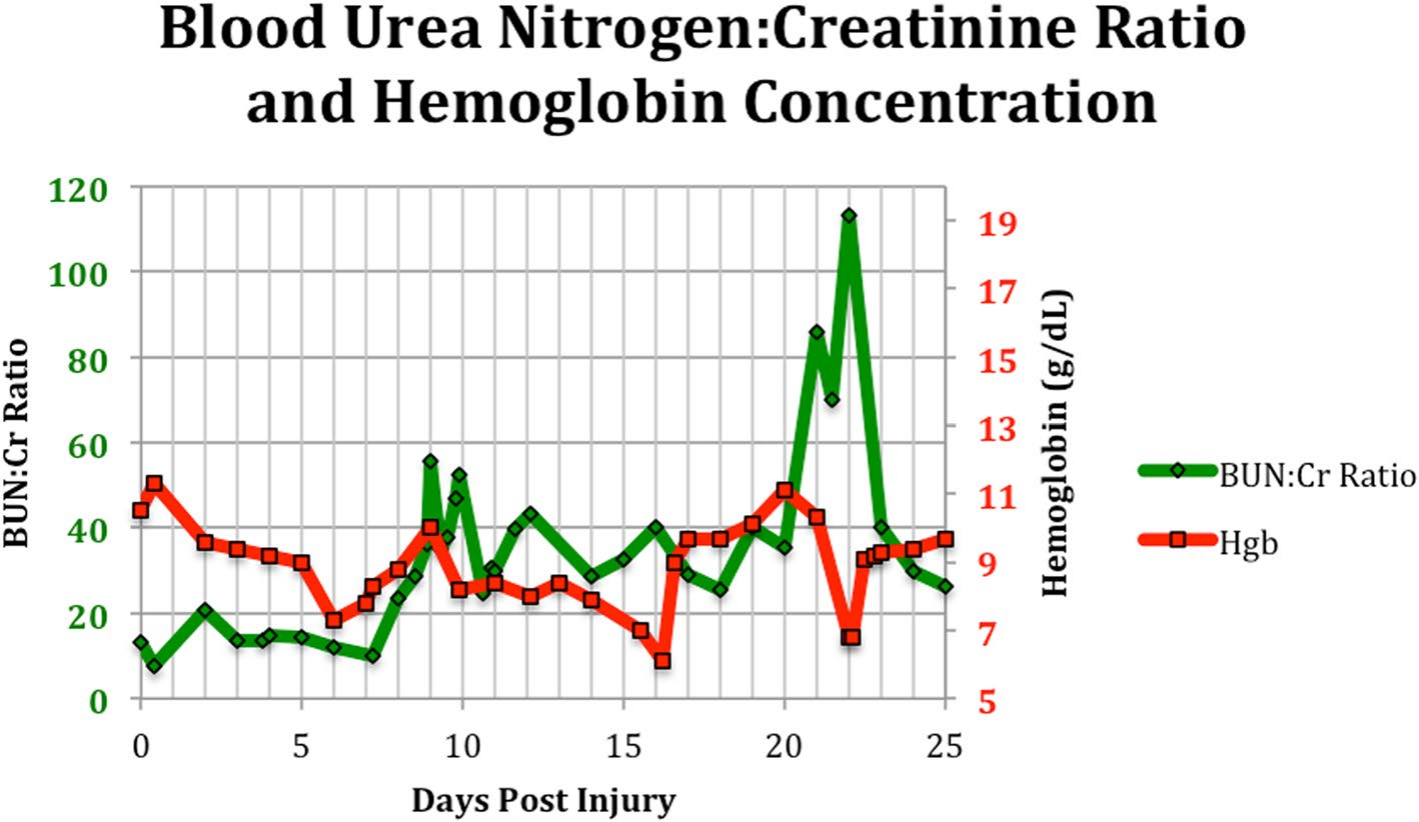
Line graph of patient BUN: Creatinine ratio alongside hemoglobin concentration, showing significant BUN: Cr spike one day before expected Hgb drop. Long School of Medicine, University of Texas Health Science Center at San Antonio, San Antonio, TX, USA

Two weeks post injury, a tracheostomy was performed and a percutaneous endoscopic gastrotomy tube was also inserted. During the latter procedure, normal gastric and duodenal anatomy was noted. The patient remained mildly anemic for the remainder of her intensive care unit (ICU) stay, with intermittent fever that eventually broke 3 weeks post-injury. On day 21 of her hospital stay, the patient’s BUN spiked from 18 mg/dL to 43 mg/dL (Fig. 1), along with a 2.5-fold increase in her BUN: Creatinine ratio, suggestive of possible GIB (Fig. 2). However, typical symptoms or signs of a recent or active bleed were absent. Hemoglobin remained stable initially, even on recheck, for 25 h following BUN spike, and neither diaphoresis nor change in stool color was noted. PEG tube was inspected by general surgery and showed no abnormal findings, suctioning of gastric contents revealed only normally colored feeding formula. The patient also denied abdominal pain or a sense of impending doom, further reassuring against a possibly significant GIB.

**Fig. 3:**
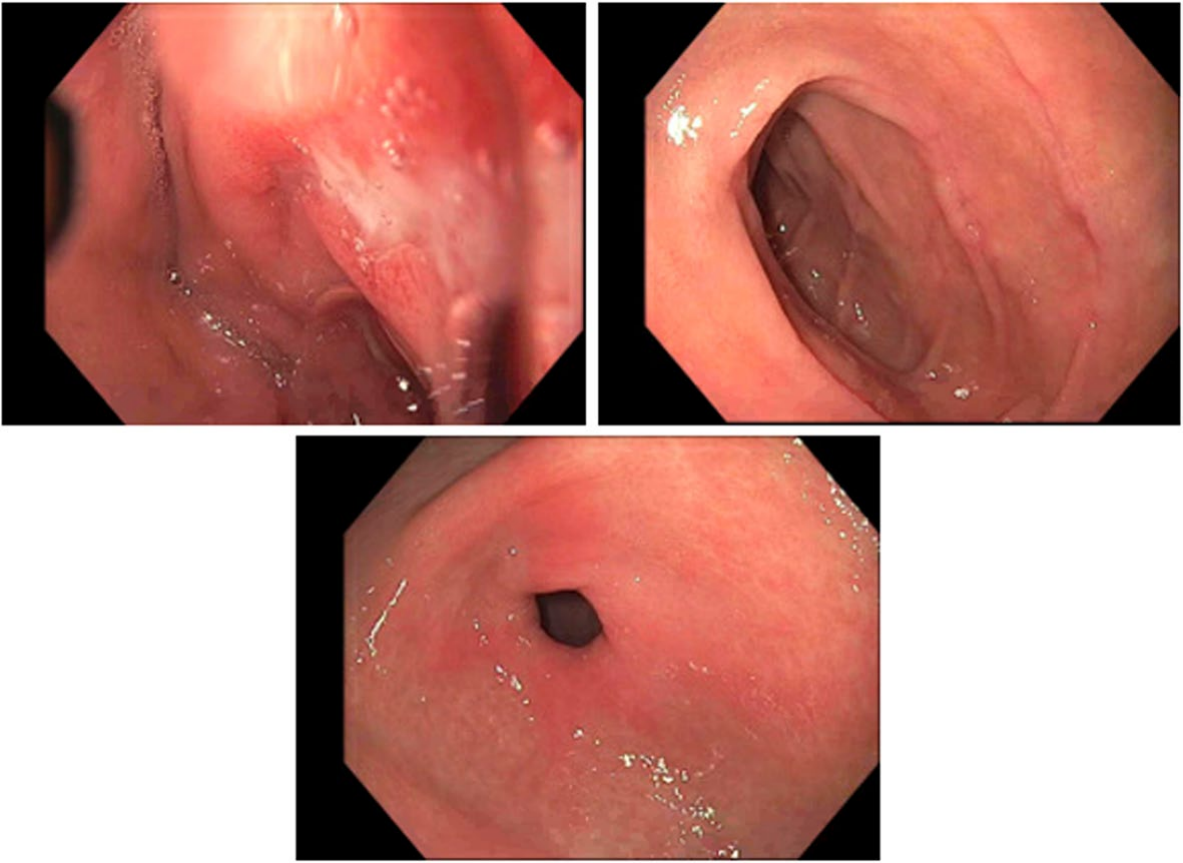
Peptic ulcers noted in the pylorus and the first two segments of the duodenum

The following day, a 3.5 g/dL drop in hemoglobin was noted while the patient remained asymptomatic, prompting a gastroenterology consult under heightened suspicion of GIB (Fig. 2). Esophagogastroduodenoscopy evaluation was performed and revealed three non-bleeding duodenal ulcers with clean bases in the duodenal bulb and sweep (Fig. 3). A biopsy for Helicobacter pylori was collected during the procedure and later interpreted as negative. No signs of active bleeding were noted at this time from the ulcer. Gastroenterology recommended an eight [8] week course of omeprazole. She received a one-time transfusion of two units of packed red blood cells (pRBCs). Large amounts of melanotic stool consistent with GIB followed that afternoon and lasted for several bowel movements.

By the evening, her hemoglobin had plateaued at 9.1 g/dL, and she remained clinically stable until her discharge to a spinal cord injury rehabilitation center. At the time of discharge, the patient remained ventilator dependent.

## Discussion and conclusions

Though GIB has historically been reported in numerous tSCI cases, our patient marks the first reported instance of dysautonomically masked gastroduodenal bleeding in acute spinal cord injury. This case highlights the importance of a watchful clinical eye in management of tSCI and exposes a window in which the patient is in severe danger of succumbing to latent pathology. It further calls for possible surveillance for GI ulceration given its significantly high incidence rate, or possibly even a change in gastrointestinal prophylactic strategies in this patient population.

In tSCI, gastroduodenal ulcerations have high incidence and are multifactorial in etiology, with significant contributions being stress of traumatic injury, mucosal ischemia, and reduced vagal tone [10]. Due to the dysautonomic and severely traumatic nature of injury in this patient population, stress-associated ischemia might have a role in impairing the mucosal defenses of the upper gastrointestinal tract against peptic ulceration [11]. Combined, these factors could explain these patients’ elevated risk for gastroduodenal ulceration.

Our patient had several predisposing factors to GIB. These included a recent procedure, steroid administration, mechanical ventilation, and prophylactic anticoagulation. These risk factors are not unique to our patient but exist in many patients with tSCI. It is worth noting that PEG has been associated with a small incidence of upper GIB, though these bleeds are mostly limited to the stomach and esophagus [12]. Two of our patient’s ulcers were found in the proximal duodenum possibly suggesting a different culprit. Historically, high dose steroids have been used early in tSCI to preserve neurological function, but current evidence doesn’t support such practice. Our patient received dexamethasone for the treatment of COVID-19 pneumonia. Some studies link high-dose steroid administration in acute tSCI with increased incidence of complications such as GIB, pneumonia, and other infections [2]. A recent meta-analysis suggested, however, a statistically insignificant association between methylprednisolone administration and GIB [9]. Overall, the risks and benefits of steroid therapy are under some debate in tSCI. Mechanical ventilation has also been strongly associated with gastrointestinal ulceration, though existing research focuses primarily on the role of physiologic dysfunction rather than ventilator support in the generation of gastroduodenal ulcers [13, 14]. In addition, enoxaparin, a low molecular weight heparin, was administrated throughout a large part of our patient’s hospital stay as a standard of care for prevention of venous thrombotic events. The anticoagulative properties of heparins impair the patient’s ability to form clots, and thus increase bleeding risk and significance. Because several factors influenced our patient’s development of GIB, we cannot assert her dysautonomia was causative, rather we put it forth as a predisposing factor. Thus, the neurological nature of her injury contributed alongside her medications and supportive care in generating an exceptionally high-risk scenario for GIB.

More importantly, we would like to draw the main attention to how delayed and masked her significant GIB presentation was. Typically, GIB presents with coffee-ground emesis/gastric content, hematemesis, melena, hematochezia, abdominal cramps, drop in hemoglobin and at times a sense of impending doom. Melena usually ensues within 4–20 h of bleeding [15]. A hemoglobin drop of 3 g/dL over several hours can provide early suspicion of such a bleed. The nuance of our case lies in how our patient’s dysautonomic condition masked her bleed by significantly and hazardously delaying symptom presentation.

Unfortunately, neurogenic bowel is a common complication of tSCI patients linked to autonomic system dysfunction, which resulted, in our case, in a delay in melena presentation and a 36-h period of almost wholly silent GIB to traditional detectors. In this interval, a rapid BUN elevation was noted early on, and served as the primary impetus for endoscopic examination of the upper gastrointestinal tract. Prior to the procedure, the patient was bleeding and thus at risk of succumbing to an illness covert to the medical care team.

While an acutely elevated BUN: Cr ratio can indicate an ongoing upper GIB, as commonly reported in the literature [16], it cannot be consistently relied upon. Besides gastroduodenal hemorrhage, several other factors in this patient population may result in this elevation including the catabolic state in acute tSCI, and acute renal failure, among other possibilities.

Concealment of acute GIB in the context of tSCI is sparsely documented in the literature. A single report of a patient with lower GI ulceration complicated by neurologic deficits associated with tSCI was found in the literature [17]. Additionally, a retrospective study from 1984 on the acute abdomen post tSCI somewhat corroborates dysautonomic masking of gut pathology, but is specifically in the context of spinal shock [18]. Timely symptomatic presentation of GIB (melena, hematemesis, etc.) relies on normal intestinal peristalsis. In a literature investigation of the effect of blood flow on catharsis, evidence suggested that even brief ischemia or hypoxia could significantly slow gastrointestinal smooth muscle contraction impeding peristaltic reaction to GIB. A recent study demonstrates ischemia, and subsequent GI dysmotility, in rat models due to decreased superior mesenteric arterial blood flow following autonomic dysreflexia associated with tSCI (19). Thus, stress-induced ischemia or hypoxia could not only be partly responsible for peptic ulceration in our patient, but also for further decreased gut motility in a system already impaired by dysautonomia, all of which delayed traditional presentation.

GIB in tSCI patients may very well be clinically silent due, in part, to dysautonomia. This presents a clinical dilemma for an already vulnerable population, especially the less closely monitored patients such as those outside the ICU or post hospital discharge. Thus, clinical diligence and hypervigilance is paramount in their care. Management of the tSCI patient calls for minimization of modifiable risk factors for GIB and potentially deployment of an alternative GI prophylaxis plan, or even a surveillance strategy for GI ulceration in tSCI given its significantly high incidence rate.

## Data Availability

Presented data is available from the corresponding author upon reasonable request.

## Abbreviations

GIB: Gastrointestinal bleed
tSCI: Traumatic spinal cord injury
BUN: Blood urea nitrogen
Cr: Creatinine
COVID-19: SARS-CoV-2 virus
PEG: Percutaneous endoscopic gastrotomy
Hgb: Hemoglobin
pRBCs: Packed red blood cells
